# Gaussian Mixture Model of Heart Rate Variability

**DOI:** 10.1371/journal.pone.0037731

**Published:** 2012-05-30

**Authors:** Tommaso Costa, Giuseppe Boccignone, Mario Ferraro

**Affiliations:** 1 Dipartimento di Psicologia, Università di Torino, Torino, Italy; 2 Dipartimento di Scienze dell'Informazione, Università di Milano, Milano, Italy; 3 Dipartimento di Fisica, Università di Torino, Torino, Italy; Université de Nantes, France

## Abstract

Heart rate variability (HRV) is an important measure of sympathetic and parasympathetic functions of the autonomic nervous system and a key indicator of cardiovascular condition. This paper proposes a novel method to investigate HRV, namely by modelling it as a linear combination of Gaussians. Results show that three Gaussians are enough to describe the stationary statistics of heart variability and to provide a straightforward interpretation of the HRV power spectrum. Comparisons have been made also with synthetic data generated from different physiologically based models showing the plausibility of the Gaussian mixture parameters.

## Introduction

Heart rate variability (HRV), the amount of fluctuations around the mean heart rate, is a valuable tool to investigate the sympathetic and parasympathetic functions of the autonomic nervous system, see, for instance [1] and references therein. In addition, heart rate variability is a key indicator of an individual cardiovascular condition and a prognostic index in the course of myocardial infarction, heart failure, diabetic neuropathy, essential hypertension, etc. [2], [3], [4]. Thus is not surprising that it has been the object of much research and that a variety of approaches have been applied to its analysis.

The normal rhythm of the heart is controlled by processes of the sinoatrial node (SA) modulated by innervations from both the sympathetic and parasymphatetic (vagal) divisions of the autonomic nervous system (ANS, a part of the nervous system that non-voluntarily controls organs and system body). ANS has central nuclei located in the brain stem and peripheral components accessing internal organs. Symphatetic and parasymphatetic systems that work as antagonists in their effect on target organs, via chemical mediators: the acetylcholine released by parasympathetic terminals slows the rate of the SA node, whereas the norepinephrine released by sympathetic terminals speeds up the SA node rhythm. The relative roles of the two systems can be determined by blocking their activity with a pharmacologic antagonist: sympathetic blockade can be obtained with guanethidine or pronethalol, parasympathetic blockade with atropine.

**Figure 1:**
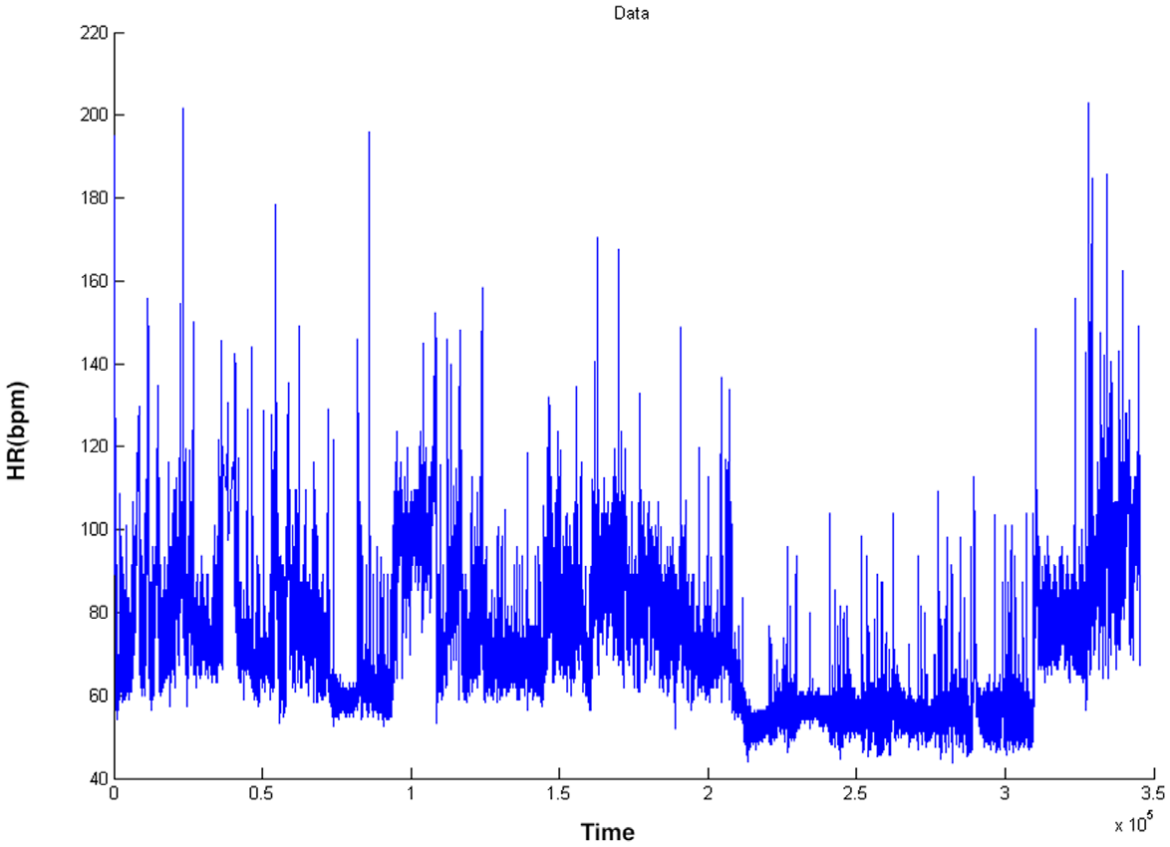
A typical 24 hour heart rate time series. Beats per minutes are shown as a function of time.

The statistical behaviour of the heart rate can be analyzed by replacing the complex waveform of an individual heartbeat recorded with the time occurrence of the contraction (the time of the peak of wave named QRS complex), which is a single number. Mathematically, the heartbeat sequence is modeled by a unmarked point process that reduces the computational complexity of the problem and allows its analysis by well known methods. Thus, the occurrence of a contraction at time 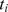 is represented by an impulse 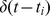 so that the heartbeat sequence can be expressed as
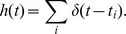
From this sequence the time intervals (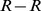 intervals) 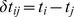, 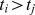 between two successive peaks can be determined, as a function of time 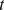; thus a new time sequence is obtained and HRV is precisely the variation of 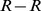 intervals. Finally time intervals are converted in beats per minute (bpm), an example is presented in Fig. 1.

In general HRV has been studied by considering statistics of 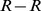 intervals (time domain analysis) or by spectral analysis of an array of 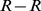 intervals (frequency domain analysis) [5], [3], [6].

Time domain statistics use linear models to calculate the overall variance or the variability between successive interbeat intervals: typically they produce short-term variability (STV) indices representing fast changes in heart rate and long-term variability (LTV) indices taking into account slower fluctuations (fewer than 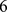 per minute). The time domain methods are computationally simple, but are not able to discriminate between sympathetic and para-sympathetic contributions of HRV that are known to operate on HR in different frequency bands.

In fact, experiments of electrical stimulation of the vagus nerve in dogs showed that vagal regulation modulates the HR up to 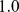 Hz, whereas symphatetic cardiac control operates only below 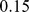 Hz [7]. In humans the parasymphatetic blockade eliminates most HR fluctuations above 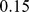 Hz, whereas the symphatetic blockade reduces HR fluctuations below 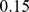 Hz leaving those at high frequency largely unaffected. Hence, HRV at high frequency (HF) components is a satisfactory, partly incomplete, index of the cardiac control, whereas low frequency (LF) components reflect both symphatetic and parasymphatetic modulation [8].

Furthermore, extensive statistical studies [6] have shown that the use of normalization of LF powers by total variance, or of the LF/HF power ratio, increases the reliability of spectral parameters (measured by the Spearman correlation) in reflecting sympathetic cardiac modulation, particularly when the cardiac sympathetic drive is activated [6]; for an in-depth discussion, see [9].

Because of this experimental evidence spectral analysis has become an increasingly popular method to investigate heart rate variability because it provides the basic information of how power distributes as a function of frequency. Spectral analysis enables to identify and measure the principal rhythmical fluctuations that characterise the 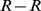 time series and contain physiological information; further, it has has been proven to provide important and accurate information on sympathetic and vagal modulation of sinus node in normal subjects and in patients with a variety of organic heart diseases, see, for instance, [1].

The main algorithm used to calculate the power spectral distribution are the fast fourier transform on uniformly resampled data and the lomb periodogram based on non uniform sampling. However the latter is not a consistent statistical estimator [10].

These methods are limited by implicit assumption of linearity and stationarity. Biological oscillators rarely meet these requirements and then it is difficult, in certain conditions, discriminate the two branches of the autonomic nervous system in a clear manner.

In this paper we argue that useful information on the role of these two systems can be gained by decomposing the signal in elementary components in the time domain, and that this can be done by determining, via some statistical procedure (namely, a greedy expectation maximization algorithm), the combination of Gaussians that best approximate the data.

Mixture of Gaussians have been used previously in an automatic classifier for electrocardiogram (ECG) based cardiac abnormality detection [11] and in frequency domain to generate realistic synthetic electrocardiogram signals [12]. Here, in a different vein, we exploit them to appropriately represent and characterize the multimodal marginal distribution of HRV series, a feature arising from non linear correlations of the time series that, in turn, are related to the peculiar physiological aspects of the neuroautonomic control of the heart rate.

### Mixture modelling of heart rate measurements

Consider a time series of heart rate measurements 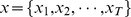, 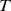 being the number of time points in the series, such as that represented in Fig. 1. Due to fluctuations of various origin [5], it can be considered as generated from a random process, where each 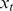 is an instance, or realization, of a random variable 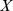. In the most simple case, one could assume that the time series is a sequence of samples 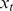 independently drawn from one known distribution 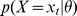, e.g. Normal or Poisson; then, the parameters 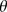 of such distribution could be easily estimated from the observed samples. Unfortunately, this is not the case as it can be simply noticed by inspecting the shape of the data histogram (the empirical distribution representing the unknown 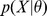), which is clearly multimodal.

Multimodality occurs because of non linear correlations of the time series and, most important, due to the multi-component structure of the physical process that originated the data [13].

To gain some insight on this issue, it is more convenient to generally describe time series models as statistical models that specify a structure of conditional dependencies on the joint distribution 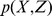, where 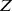 is a latent variable or hidden state variable.

Conventional time series models are global models. They can be linear, assuming that the next value 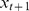 is a linear superposition of preceding values [14], or they can be nonlinear. For instance, nonlinear autoregressive processes (NLAR) have been widely used [15], [16]; these models assume, in the simplest case (first order model) that it is possible to generate 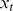 at time 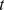 by taking into account its conditional dependence on the previous value 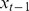 and on current state 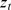, namely 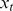 is sampled as 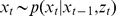 where 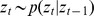, the latter specifying the Markov dynamics of state transitions. Such single, global, and traditionally univariate models are well suited to problems with stationary dynamics. However, the assumption of stationarity is violated in many real-world time series, such as HRV series. An important sub-class of nonstationarity is piece-wise stationarity (also called stationarity by parts and multi-stationarity) where the series switches between different regimes; in this case, state-space models with switching dynamics (multiprocess dynamic linear models) can be exploited. Typically, in switching models a discrete switching random variable 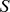 is introduced, so that the state dynamics 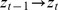 depends on the sampled regime, for instance 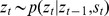 where 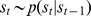.

**Figure 2:**
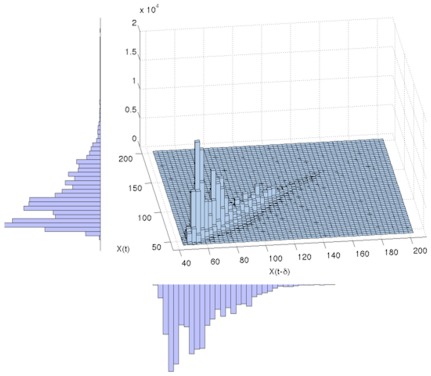
A bivariate histogram computed from the HRV time series, which approximates the joint density 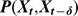, where 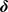 is the time lag (in the example 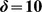). The univariate histogram on the left stands for the marginal distribution 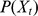.

Different variations can be constructed from this basic models (see [13], for detailed discussion). But what is interesting, from the standpoint of this work, is that both nonlinear autoregressive processes and switching state-space models give rise to joint distributions of lagged data, 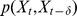, 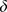 being the time lag, whose marginal distribution 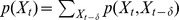 is a multimodal distribution. An example is provided in Fig. 2 in terms of empirical joint density distribution (bivariate histogram) of a HRV time series at lag 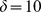.

Thus, by modelling the multimodal marginal distribution 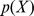 of HRV data, it is possible to achieve useful insights on the process that generated the data. For example, a similar approach has been addressed in the field of solar radiation models [13], where the two modes in the distribution of the radiation time series were shown to be produced by cloudy times, when radiation is indirect, and cloud-free times, when radiation is direct. In the same vein, a similar application has been reported in [17] to distinguish physical regimes underlying equatorial Pacific sea surface temperature data, and for modelling BOLD signals in fMRI [18].

For modelling complex multimodal probability distributions, mixture models are widely used. Taking a generative view, a data sequence 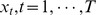 can be sampled from a mixture model by iterating the following two steps:

1. sample which component 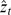 among the 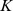 available is going to generate the data:
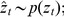
(1)
2. sample the actual data 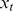


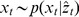
(2)

Here 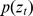 and 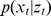 are Multinomial distributions, respectively; by using a 1-of-K representation for the state variable 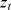, namely 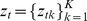 and 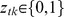, that is 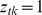 indicates that 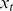 has been generated from the 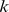-th mixture component,
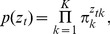
(3)with 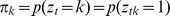 representing the prior probability of choosing the 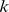-th component and
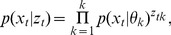
(4)where 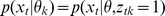 is the 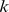-th component distribution characterized by parameters 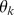.

It is easily seen that the marginal distribution 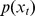 can be written in terms of the linear combination of some number 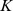 of simpler, component distributions by marginalizing the joint distribution 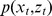 over all possible states of 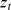:
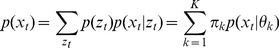
(5)where probabilities 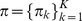 are named in this linear superposition representation the *mixing coefficients*, satisfing 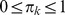 and 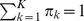.

In particular, for modelling arbitrary multimodal marginal distributions, Gaussian or normal components have been widely used:
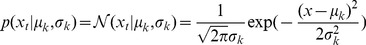
(6)here parameters 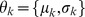, in case of univariate components, denote the mean and the variance of the 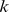-th Gaussian component, respectively.

Learning the mixture, namely, estimating the weights 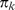 and the parameters 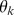 of each component, can in principle be carried out through maximisation of the likelihood with respect to such parameters, or more conveniently by maximizing the log-likelihood
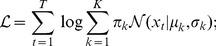
(7)the latter is difficult to optimize because it contains the logarithm function of the sum. A suitable method to perform log-likelihood maximization of a mixture is the Expectation-Maximization (EM) algorithm [19].

The EM algorithm is simple to implement although it suffers from known limitations: there is no widely accepted good method for initializing the parameters; due to its local nature, it can get trapped in local maxima of the likelihood function; further, it assumes a known number 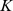 of mixing components, an assumption that does not hold for the work presented here.

To overcome the model selection problem one could resort to conventional approaches based on cross-validation that are computationally expensive, are wasteful of data, and give noisy estimates for the optimal number of components. A fully Bayesian treatment, based on Markov chain Monte Carlo methods for instance, will return a posterior distribution over the number of components. More viable solutions are variants of the Variational Bayes Expectation-Maximization algorithm [20] that require the introduction of continuous hyper-parameters whose values are chosen to maximize the marginal likelihood, or more complex procedures currently under study in the field of nonparametric Bayesian methods such as Dirichlet Process Mixtures under the assumption of an infinite mixture model [21], [22].

More simply, we have adopted a greedy variant of the EM algorithm [23]; [24]. An important benefit of the greedy method, compared to the previous ones, is the production of a sequence of mixtures, which resolves the sensitivity to initialization of state-of-the-art methods, and has running time linear in the number of data points and quadratic in the final number of mixture components; also, it facilitates model selection.

The basic idea is straightforward: instead of starting with a random configuration of all components and improve upon this configuration with EM, the mixture is built from one initial component by iteratively adding new components obtained through a splitting of older components. More precisely, by starting with the optimal one-component mixture (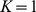), whose parameters are trivially computed, following steps are repeated until a stopping criterion is met: 1) find a new optimal component 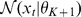 and the corresponding mixing parameter 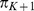 so that the log-likelihood embedding the 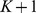 components
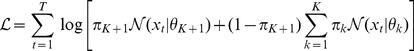
(8)is maximized with respect to parameters 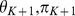; 2) set the new mixture as
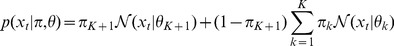
(9)and let 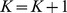; 3) update the new mixture 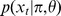 of 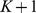 components using EM;

In step 2), dealing with the insertion of a new component, the method constructs a fixed number of candidates per existing mixture component; the candidate that maximizes the log-likelihood when mixed into the existing mixture is retained (for details see [24]).

The method stops the partial updates if the change in log-likelihood of the resulting 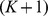-component mixtures drops below some threshold or if some maximal number of iterations is reached, or if a desired number of components 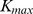 is obtained (for instance, along experiments we set 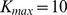, which was in practice never reached).

Clearly, the stopping criterion could be any model complexity selection criterion (like Minimum Description Length, Akaike Information Criterion, Cross Validation, etc.), so that the optimal number 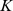 of components is automatically determined. However, an advantage of the greedy method is that it produces a sequence of mixtures that can be used to perform model complexity selection as the mixtures are learned. In particular a kurtosis-based selection criterion, like the one in [25], can be used here.

## Results

Experiments have been conducted on both real data and synthetic data. Real data analysis was performed on ECG recordings collected with the procedure described in the section on material and methods. Analysis of synthetic data generated by using well known models of physiological aspects of the neuroautonomic control of the heart rate, [26]–[27], has been aimed to further verify the physiological plausibility of the Gaussian mixture parameters learned via the Greedy EM algorithm. The rationale behind this analysis is that synthetic data obtained from models governed by such parameters should be consistent with the experimental ones.

### Real data

**Figure 3:**
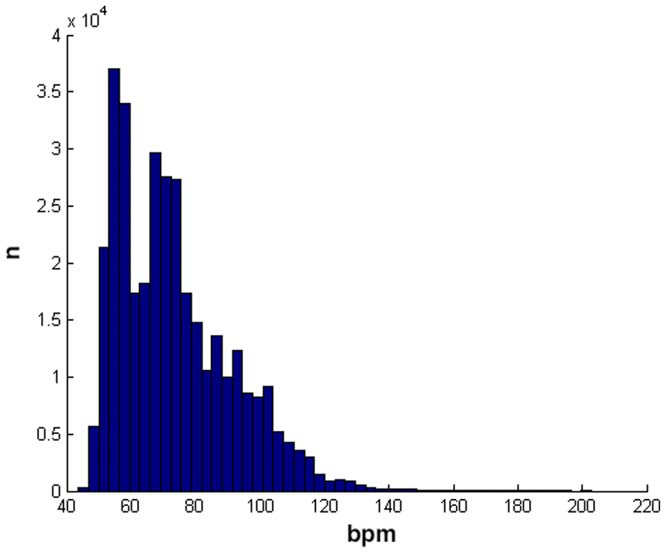
An histogram of a 24 hour heart rate time series, showing the number of occurences of bpm values.

A typical time series of heart signals is displayed in Fig. 1 and the corresponding histogram is shown in Fig. 3.

**Figure 4:**
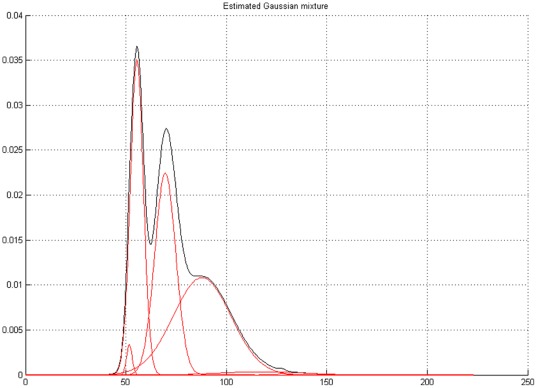
Gaussian Mixture Model of a 24 hour heart rate. Here the components corresponding to the 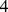 largest weights are presented. Note that the fourth weight is much smaller than the others 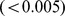. The red lines represent the gaussians multiplied by their weights and the black curve the result of the mixture.

Finally Fig. 4 presents the results of the analysis, where each gaussian is multiplied by its weight: here only four components are shown, three with weights larger than 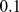, the fourth being less than 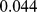. It is apparent from the figure that just the first three components are important in determining the mixture.

It should be noted that heart rate is positive definite, whereas Gaussians may assume negative values: however, by inspection of the location of the data from the marginal distribution and the related fitting obtained through the Gaussian mixture model learned from HRV data, the probability of generating negative data is negligible.

**Figure 5:**
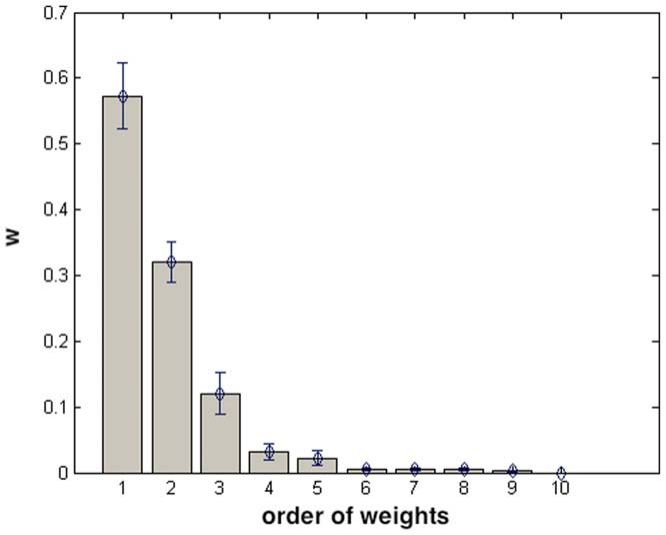
Values, averaged over time series from 120 subjects, of the weights as determined by the algorithm, in decreasing order of magnitude. Bars indicate the standard deviation of the mean (error bars).

The relevance of just three weights is not limited to individual recordings, but it is confirmed by the averages, over all subjects, of weights values, shown, in decreasing order of magnitude, in Fig. 5.

**Figure 6:**
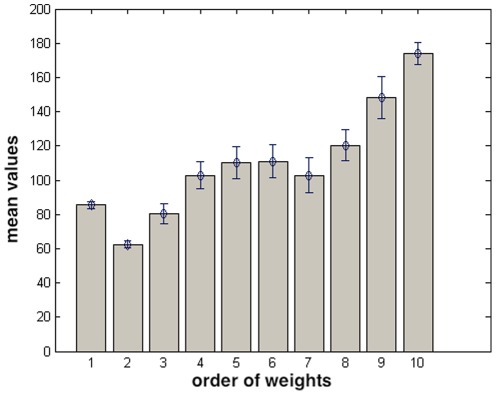
Averages, over time series from 120 subjects, of mean values of gaussians, as a function of the order of the weights. Bars are the error bars.

Further information on the structure of 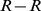 signals can be gained by considering mean and variances of the Gaussians. The trend of the means plotted in the order of decreasing weights is almost monotonically increasing, see Fig. 6, the first three components of the mixture having the smallest mean values. This shows that components with beat/minute values larger than 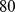 play no significant role in the determination of 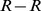 intervals.

**Figure 7:**
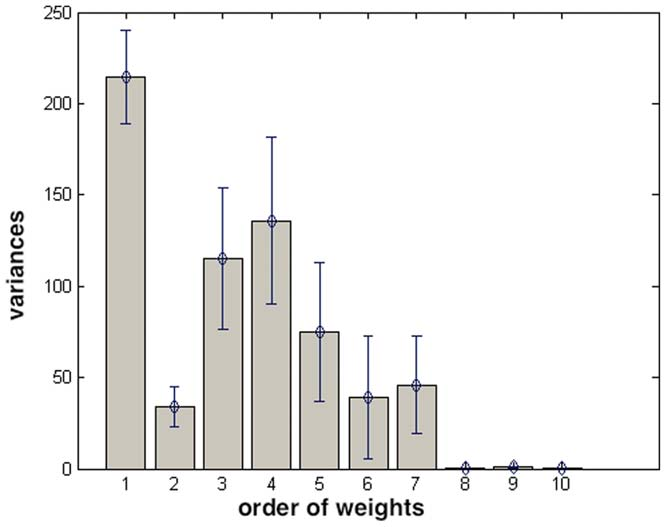
Variances of Gaussians, averaged over time series from 120 subjects, as a function of the order of the weights. Bars indicate the standard deviation of the mean (error bars).

Variances do not show a definite trend, see Fig. 7, but it should be noted that the first component has by far the largest variance (almost by a factor 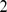). That means that its values extend on a large part of the rate interval and therefore it gives (by far) the largest contribution to the power spectrum of the signal.

**Figure 8:**
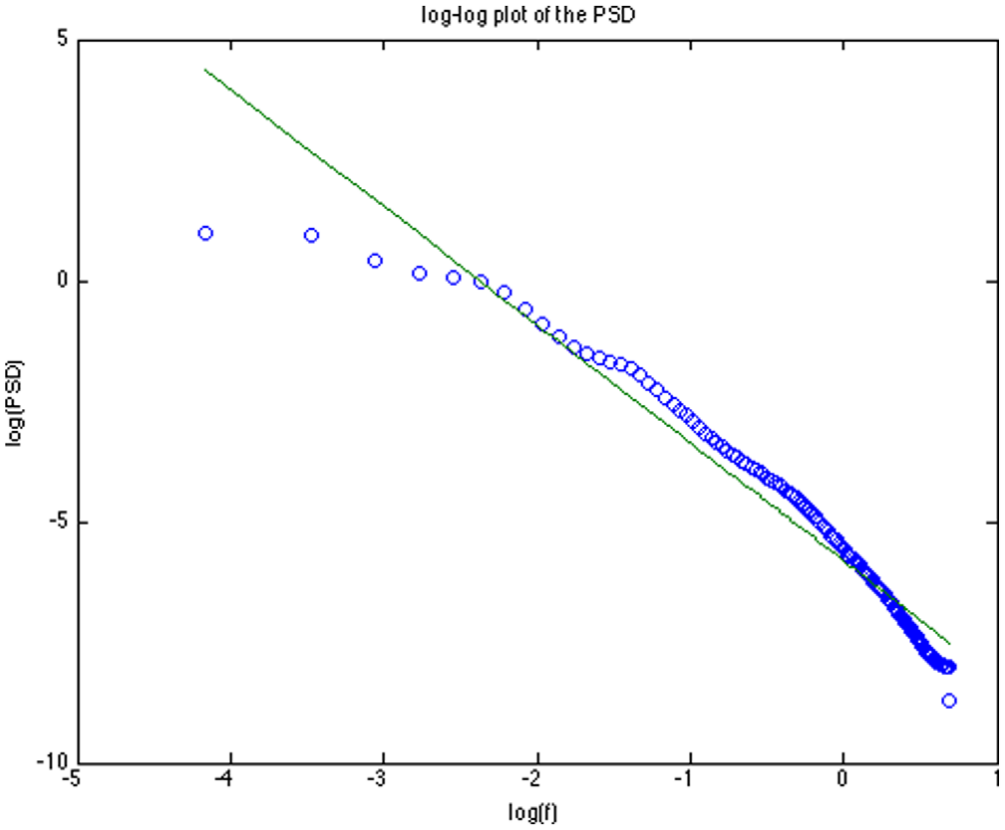
Power spectrum, averaged over 120 subjects, of the heart rate time series, over 24 hours records. The scale is log-log. The slope, 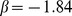 is computed in the range 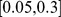.

As a test, we have computed the power spectrum of the time series, averaged of all subjects: it shows, in the range 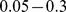 Hz the well known 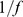 trend that has been observed in several studies and has been ascribed to complex mechanisms such as intermittency [28] and self-organized criticality [29], see Fig. 8.

The same trend can be obtained from the power spectrum of a time series of 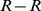 signals generated by applying the sampling procedure specified via Eqns. (1), (2), (3) and (4) and by using just the three most relevant Gaussians as derived from the data.

In conclusion, heart rate variability can be explained by a mixture of just three Gaussians; what remains to be investigated is the relation between the Gaussian components and the action of sympathetic and parasympathetic systems.

As remarked in the Introduction, there is an ample evidence that the dynamics of sympathetic and parasympathetic systems occurs in different frequency bands. Now, if a specific Gaussian captures the action of one of the two systems, keeping in mind that the spectrum (PSD, power spectral distribution) represents the contribution to variance of the different frequency bands [30] one should expect a correlation of the PSD at the three bands of very low, low and high frequency with the variance of three gaussians calculated for the time series of each subject.

The results, reported in table 1, show significant correlations (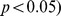 between the variance of the first two gaussian and the power spectrum of the low and high frequency bands, respectively. This suggest that the two gaussians with the largest weights are related to the activation of the symphatetic and parasymphatetic component of the autonomic system.

**Table 1:** Correlation analysis results.

Measures	LF	HF
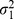	0.37	n.s
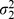	n.s	0.25

Significant correlations (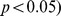 between the variance of the first two Gaussian and the PSD of the low and high frequency bands.

### Synthetic data

The fact that just three Gaussian components of the signal are enough to explain most of the variability of heart rate, suggests that they may correspond to the three major inputs, namely those coming from the sinoatrial node, responsible for the initiation of each heart beat, and from the parasympathetic and sympathetic branches of the autonomous nervous system. If this is the case our results should be reproduced by models that make variability of heart rate to depend on the activity of only these three inputs.

Such is the case, for instance, when a simple model is used adapted from the well know class of integral pulse and frequency modulation models (IPFM) [31]. In IPFM the input signal is integrated until a threshold 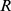 is reached at which a pulse is generated at time 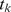; the integrator is then set to zero and the process is repeated. The general form of the IPFM model is
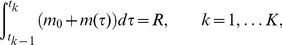
(10)where it is assumed that 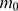 is a term accounting for the sinoatrial node and 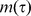 is the input signal representing the autonomic activity, described as
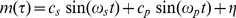
(11)where 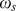 and 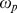 are the frequencies of the oscillators describing the sympathetic and para-sympathetic branches of the ANS, 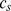 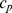 are weights and 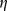 is Gaussian noise. We have used this model to simulate large samples of HRV records and these synthetic data have been eventually analyzed with the same algorithm used for the experimental data.

Gaussian mixture modelling produced just three Gaussians with weights larger than 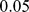; furthermore their values and those obtained from experimental data are not significantly different (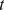
- test, 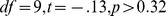).

These results may be not surprising since the model contains explicitly neural oscillators, thus as a further test, we have used a quite different type of model proposed in [27] and [32], where changes in the interbeat interval 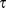 are described by:
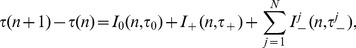
(12)where 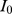, 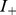 and 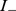 are inputs coming the sinoatrial node, the parasympathetic and sympathetic fibres, respectively, whereas 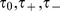 are time constants.

Each of the inputs in (12) is given the form
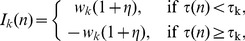
(13)where 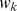 is the strength of the feedback input biasing 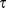 to return a preferred level 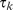, and 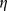 represents uncorrelated noise. In turn 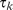 are random step-like function of time drawn from an uniform distribution and constrained within a certain interval. (see [32], [27] for further details). From a statistical standpoint, this model can be seen as a state-space model with switching dynamics (see discussion in Section 2).

Statistical analysis on large samples of simulated data shows again that the Gaussian decomposition yields just three weights larger than 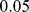, and that there is not significant difference from those obtained from the empirical data (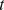-test, 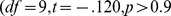).

It is well known that there exist several factors affecting heart rate, for example see [9], but what these models show is that in HRV data the main component derived by the gaussian mixture can be well described by the three major inputs that influence the heart rate: symphatetic and pharasymphatetic control plus the oscillation of the sinoatrial node.

Thus our results further support the evidence of a major role of these three components in producing the variability observed experimentally.

## Discussion

In this paper we have presented a novel method to analyze heart rate variability, based on a Gaussian mixture decomposition of the signal. This approach presents several advantages: first, given enough Gaussian components, mixtures can approximate arbitrary complex distributions and the mixture model covers the data well (dominant patterns in the data are captured by component distributions).

Furthermore, the use of gaussians allow a straightforward interpretation of the properties exhibited by the power spectrum.

In addition well-studied statistical inference techniques are available to determine the parameters of the mixture, that here have been learned via maximum likelihood in a greedy fashion, namely, by incrementally adding components to the mixture up to a desired number of components 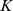.

Results show that just three Gaussians (i.e., 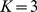) are enough to predict heart rate variability, and that the mean and variance values of the relevant components are coherent with physiological measurements.

Means of the main components provide a lower bound of the beat/minute values relevant in the formation of 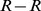 time series, while variances supply a link with frequency structure of the signal. This link has been used in a correlation analysis whose results suggest a possible identification of the activity of the different branches of the ANS with the components of the Gaussian mixtures.

Finally we have also found that the decreasing trend 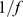, observed in the data, can be derived by using the learned Gaussian mixtures as a generative model. This result is relevant because it is a further evidence that this approach indeed extracts the relevant structure of the process.

Most often probabilistic models cannot explain by themselves the physical processes generating the data, one exception being the kinetic-molecular movement within a gas. Indeed the physics of the phenomenon under study can be accounted for by models involving solution of the appropriate governing equations.

In this perspective, we have investigated the relation of this probabilistic model with well known models used in the literature [26]–[27] to simulate the action of sinoatrial cells, and sympathetic and parasympathetic systems. The results show that the parameter learned from the data when plugged in dynamic model produce synthetic data consisten with real ones.

## Materials and Methods

### Participants

A hundred healthy volunteers, 50 males and 50 females, (age range 18–40, average 24.73, SD 4.35), took part in the recording session. They had no history of cardiac injury or psychological diseases and all took part voluntary and gave an informed consent. Prior to the studies, they were acclimated to the settings, and practiced with the apparatus. They refrained from alcohol or caffeine intake and strenuous physical activity for 12 h preceding the study sessions. All of the participants gave their informed written consent, in line with the Declaration of Helsinki, and the study was approved by the Ethic Committee of the Department of Psychology, Turin University.

### Procedure

Electrocardiogram recordings were obtained using a Holter Lifecard CF (Del Mar Reynolds Medical Ltd.). Each participant was asked to wear the Holter for 24 hours and to come back the following day at between 5 and 6 p.m. to return the device. During debriefing, a researcher checked the apparatus and asked further questions as necessary.

### Data reduction

The QRS detection and arrhythmia analysis were performed using a DelMar Avionics arrhythmia analyzer (Impresario). No arrhythmia was detected in the data analyzed. The presence of artifacts was checked manually, although no abnormalities were found in any subject. The 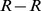 intervals were then calculated as the time interval between two consecutive R-waves.
